# The vitamin D level in umbilical cord blood in premature infants with or without intra-ventricular hemorrhage: A cross-sectional study

**Published:** 2018-07

**Authors:** Hassan Boskabadi, Maryam Zakerihamidi, Raheleh Faramarzi

**Affiliations:** 1 *Department of Pediatrics, Faculty of Medicine Sciences, Mashhad University o Medical Sciences, Mashhad, Iran.*; 2 *Department of Midwifery, School of Medicine, Islamic Azad University, Tonekabon Branch, Tonekabon, Iran.*

**Keywords:** Premature infants, Vitamin D, Cerebral intraventricular hemorrhage

## Abstract

**Background:**

Intra-ventricular hemorrhage (IVH) is acute cerebral complications of premature infants which may lead to the long-term problems.

**Objective:**

According to the role of vitamin D in the stability of the blood vessels, the present study was carried out in order to compare the vitamin D level in the premature infants with or without IVH.

**Materials and Methods:**

This cross-sectional study was carried out on 180 premature infants in the Ghaem Hospital, Mashhad, Iran 97 infants without IVH (53.9%) and 83 with IVH (46.1%) through convenience sampling technique in 2015-2017. Serum vitamin D level of funiculus in the two groups was compared. A researcher made questionnaire was used which includes infants’ personal and laboratory information; and their mother's information.

**Results:**

Seventy nine percent of infants suffered from vitamin D deficiency in which 33.9% had a severe deficiency (less than 10 ng/ml), 30% moderate deficiency (10.1-20 ng/ml), 15% slight deficiency (20.1-30 ng/ml) and 21.1% had normal vitamin D (>30.1 ng/ml). Vitamin D mean±SD of infants in the control group, 23.71±12.98 ng/ml and case group 15.92±10.27 ng/ml (p<0.001). In total 92.8% of infants with IVH had levels of vitamin D below30 ng/ml, while this rate was 67% in infants without IVH.

**Conclusion:**

Vitamin D deficiency in the premature infants is very common. Also, the serum vitamin D level in infants with IVH was less than infants without IVH. Therefore, the recommendation of vitamin D may be effective in the prevention of neonatal IVH.

## Introduction

Intra-ventricular hemorrhage (IVH) is the most common intracranial hemorrhage in very low birth weight infants (1). Multifactorial IVH pathogens include; the prematurity of the germinal matrix, fluctuations in cerebral blood flow, hypoxic-ischemic brain damage and infants’ developmental abnormalities of hemostatic (2). Eighty to ninety percent of IVH occur from the first-day till the third day of life (3). 

The best method of screening is a cranial ultrasound on the 3-7 days of life (4-6). Severe and moderate IVH can lead to the long-term disability, cerebral palsy, mental retardation, seizures, behavioral and cognitive disabilities and death (1, 4). Premature infants are not able to regulate the cerebral blood flow and keeping brain blood pressure due to hypotension and decreased cardiac output after birth, especially on the first day of life for adapting to life outside the uterus. In addition, the reaction of cerebral vascular and self-regulatory mechanisms in the brain of premature infants, also is weak. Therefore, by decreasing the gestational age, the amount of brain self-regulatory pressure will also decrease (7). Moreover, cerebral vascular endothelium in the premature infants is very susceptible to the hypoxia. Thus, hydrostatic and osmotic changes lead to the rupture of the blood vessels (8). 

Except prematurity, the possibility of IVH increase by some factors such as; low birth weight, respiratory distress, damages caused by hypoxia, ischemia, decrease or increase of blood pressure, increase of venous pressure, pneumothorax and hypovolemia (1, 4, 9), natural delivery, long-term mechanical ventilation, and low Apgar at the fifth min (9-11). Changes in the cerebral blood flow due to self-regulation cerebral vascular disorders, decrease or increase of the cerebral blood pressure, coagulopathy, infections and lack of protective mechanisms in the cerebral vessels are involved in the occurrence of IVH (12). As a result, the rapid growth and development of the fetus, especially bone osseous calcification at late pregnancy, lead to the possibility of vitamin D deficiency in the pregnant women, and since the fetus and infant are dependent on the amount of vitamin D in the blood and breast milk, the presence of sufficient supplies of vitamin D is very vital (13, 14). 

The serum 25-hydroxy vitamin D level has been reported less than 25 ng/ml during pregnancy in 32-42% of Hindi (15) and 84% of Iranian women (16). Such a wide deficiency undoubtedly has harmful effects on the health of the pregnant women and infants (17). Since the effects of vitamin D on the vessels is reducing inflammation, angiogenesis and proliferation of vascular smooth muscle cells (18-20), and it directly affect the vascular endothelium stability (21), thus, it is likely that vitamin D deficiency plays a role in the neonatal IVH. 

Therefore, according to the long-term and serious consequences of IVH in the infants, and the possible role of vitamin D in vascular strength and preventing IVH, the present case-control study was carried out in order to compare the amount of vitamin D level in funiculus of the infants with IVH and without IVH.

## Materials and methods

This cross-sectional study was conducted on 230 premature infants with gestational age less than 37 wk in the delivery room and neonatal intensive care unit, Ghaem Hospital, Mashhad, Iran was carried out through convenience sampling method during 2015-2017.

Case group infants consist of premature infants who had brain intra-ventricular hemorrhage based on their ultrasound during third to seventh days after their birth. Control group consists of infants whose blood culture was reported negative and had a normal ultrasound and did not need resuscitation in the delivery room. Infants with perinatal infection, congenital malformations, and maternal history of anti-epileptic drugs have been excluded from the study. Samples containing 1.5 cc blood of umbilical cord that w taken at the birth from infants with birth weight less than 1500 gr and gestational age less than 32 wk. These samples were centrifuged and its serum was kept at -20^o^C and sent to the laboratories to evaluate. 

Vitamin D level was measured by Elisa Reader RT2100c, from Germany and Elisa Washing device through ELISA method. Brain ultrasound was taken for checking IVH at the 3-7 days of birth. Diagnosis and grading IVH was based on Papile criteria and was determined as follows: hemorrhage grade 1 consists limited hemorrhage to the germinal matrix, grade 2 consists intra-ventricular hemorrhage without ventricular dilation, grade 3 includes intra-ventricular hemorrhage with ventricular dilation and grade 4 consists intra-ventricular hemorrhage with parenchymal hemorrhage. IVH grade 1 and 2 are considered as the mild ones and grade 3 and 4 as the severe one (22).

By using researcher made questionnaire, personal information of infants such as age, birth weight, duration of oxygen therapy, first min Apgar score, fifth min Apgar score, duration of mechanical ventilation, the reason of hospitalization, IVH grades and laboratories’ information of infants such as WBC, sodium, calcium, Be1, PO2a, Ph1, hematocrit, Cr, Urea, blood cultures, umbilical cord vitamin D and maternal information such as age, gestational age in both groups were evaluated. Serum vitamin D level was compared between both groups with/without IVH. The amount of vitamin D was divided into 4 classes: severe deficiency (25 hydroxy vitamin D level less than 10 ng/ml), moderate deficiency (10.1-20 ng/ml), mild deficiency (20.1-30 ng/ml) and normal vitamin D (>30.1 ng/ml). Finally, IVH incidence in both groups was compared with the normal level of vitamin D and the decreased level.

**Ethical consideration**

This study was approved by the Research Committee of Mashhad University of Medical Sciences (code number: 951630.) and written satisfaction was taken from their parents before doing the research.

**Statistical analysis**

After data collection and recording information in SPSS (Statistical Package for the Social Sciences, version 19.0, SPSS Inc, Chicago, Illinois, USA), by using tables, charts, and statistical indices, the study was evaluated. To analyze and examine the relationship between the variables after normality control, independent T-test was used and for analyzing the relationship between variables with nominal scale Chi-square test was used. Analysis of covariance was used to control the variable of gestational age. p<0.50, in this study, was the significant level minimum.

## Results

A total of 33 infants were excluded from control group (16 received developed resuscitation, 11 had early-onset infection, and 3 infants’ mothers took phenobarbital and 3 had congenital anomalies). 97 infants out of 180 in the control group were without IVH and 83 infants were with IVH in the case group. In the brain ultrasound, 97 infants (53.89%) were normal, 60 (33.33%) with IVH1, 16 (8.89%) with IVH2, 4 (2.22%) with IVH3 and 3 infants (1.67%) with IVH4. 92 infants (54.76%) were boys and 76 (45.24%) were girls. 60 infants (37.73%) were born by vaginal delivery and 99 (62.26%) by the Caesarean method. 

The assessment of the vitamin D amount in our study indicated that 78.9% infants suffered from vitamin D deficiency in which 33.9% had a severe deficiency (less than 10 ng ml), 30% moderate deficiency (10.1-20 ng/ml), 15% mild deficiency (20.1-30 ng/ml) and 21.1% had normal vitamin D (30.1-45 ng/ml). Following items were studied within both groups in which there was no significant difference: mother age (p=0.704), birth weight, gestational age, duration of oxygen therapy, White Blood Cell, Na (p=0.354), Ca (p=0.754), Base exes (p=0.411), PO2 (p=0.930), pH (p=0.143) and platelet count (p=0.074). (p>0.05, Table I). There was a significant difference in both groups between the following items: hematocrit, cord blood level vitamin D (p=0.007), duration of mechanical ventilation, urea (p=0.006), and Cr (p=0.013). This means that in infants with IVH, hematocrit and serum vitamin D were lower and duration of mechanical ventilation, Cr, and urea were higher (Table I).

The most common reason of control group’ hospitalization was prematurity (42.55%) and in infants, with IVH it was RDS (40.51%) (p=0.001). Positive blood culture cases were in the infants group with IVH (7.25%) but in the control infants, all blood cultures were reported as negative (p=0.012). 63.41% of IVH infants were boys (p=0.028). The most common type of respiratory support was nasal SIMV in the Case group (69.44%) (p=0.000). There was no significant difference between delivery type in both group (p=0.101) (Table II).

**Figure 1:**
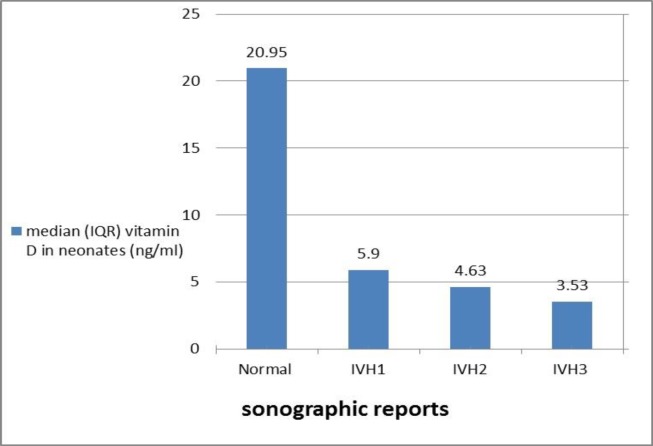
Comparison of serum vitamin D level in different grades of IVH

The serum umbilical cord vitamin D level in infants without IVH was higher than infants with IVH. Also with increasing IVH intensity, there was a decrease in the vitamin D median (Figure 1). 

**Table I:** Comparison of infant and maternal variables mean in both groups with/without IVH

**Variables**	**without IVH**	**with IVH**	**p-value**
Birth weight	1208.10 ± 222.10	1226.70 ± 280.92	0.635
Gestational age (wk)	30.00 ± 2.75	30.00 ± 2.50	0.651
Duration of mechanical ventilation (hr)	6.58 ± 6.71	11.94 ± 8.53	0.004
Duration of oxygen therapy (day)	6.39 ± 8.58	9.67 ± 7.78	0.087
WBC	11.42 ± 4.93	11.22 ± 7.43	0.931
Hematocrit	45.16 ± 3.54	40.80 ± 5.71	0.010

Data presented as mean±SD. Student t-test

WBC: White blood cells

IVH: Intraventricular hemorrhage

**Table II:** Comparison of some neonatal variables in both groups with/without IVH

**Group variables**		**without IVH**	**with IVH**	p-value*
Gestational age				
	26-30 wk	70 (72.2)	65 (78.3)	0.342
	30.1-36 wk	27 (27.8)	18 (21.7)	
Blood culture				
	Negative	97 (100)	76 (92.75)	0.012
	Positive	0 (0)	7 (7.25)	
Sex				
	Boy	45 (46.51)	52 (63.41)	0.028
	Girl	52) 53.49)	31 (0.45)	
Delivery type				
	Normal delivery	34 (32.22)	34 (44.93)	0.101
	Cesarean/Section	63 (67.78)	49 (55.07)	

Data presented as n (%).

p-values<0.05 are considered significant. Chi-square test.

## Discussion

Based on the results of this study, although the gestational age and infants’ weight were homogeneous in both groups, serum vitamin D level of funiculus in the premature infants with IVH was certainly lower than infants without IVH. To the extent that investigators searched, it is the first study which examines such an idea. High vascularity of germinal matrix and its vascular fragility can increase bleeding in comparison with areas with fewer vessels in the brain, but this can’t explain the thoroughly into-ventricular hemorrhage mechanism. Another proposed mechanism is cerebral blood flow disorder through cerebral blood flow fluctuation, which leads to higher cerebral vein pressure and abnormal blood pressure. In addition, premature infants’ coagulation system insufficiency also is effective (23). 

Srinivasan and co-worker showed that cord blood vitamin D levels are low in preterm infants and Infants with low levels of vitamin D have a higher incidence of sepsis, and ROP (24). Also, premature infants are at high risk for vitamin D deficiency and since vitamin D affect directly the vascular endothelium stability (21), possibly this vitamin deficiency can be effective in the occurrence of intraventricular hemorrhage in the premature infants. In this study, by increasing the IVH intensity from grade 1-3, the amount of vitamin D decreased inversely. 72.3% of case group infants had IVH1; 19.3% had IVH2; 4.8% had IVH3 and 3.6% had IVH4. In Abdi and co-worker’s study, the frequency of IVH1 and IVH3 were 37.5% and 3.7% respectively. By increasing the IVH intensity from grade 1-2 and 2-4, the amount of vitamin D decreased inversely (25). 

In the present study, by increasing the birth weight, the possibility of IVH became less, but weight in infants in both groups with/without IVH, had no significant difference, thus, these two groups were homogenous in this term. In Badiei’s study, the higher weight of infants at the birth time was considered as the protective factor against IVH (26). In Khodapanahandeh co-worker’s study, infants’ weight at the birth time was associated with high-grade IVH occurrence (3). In a study, birth weight was considered as risk factors and predictors of bleeding inside the brain. So that by increasing birth weight, relative chance of IVH will be decreased (27). 

In the present study, increasing the gestational age decreases the Intra-ventricular hemorrhage, but the gestational age was homogeneous in both groups. In Gleissner co-worker’s study, the risk of IVH occurrence was related to the gestational age. Infants who were born before the 28^th^ wk were at the high risk of IVH (28). In Badiei’s study high gestational age was protective factors against IVH (26). In Jalali co-worker’s study, infants with the gestational age below 35 wk had the highest possibility of bleeding than the gestational age more than 35 wk (27). As the gestational age is the most important risk factor of IVH and case group was homogeneous in this term, so, there is a possibility that decreasing vitamin D is predisposing factors in IVH occurrence. In the present study, the amount of Infants’ hematocrit with IVH was less than this in infants without IVH. Kornacka’s study showed that infants with moderate and severe IVH had an abnormal amount of hematocrit, in 50-60% of cases, at the 1 hr of their life (29). 

In Khodapanahandeh study, there was a significant relationship between low hematocrit during the first 24 hr of life and higher incidences of IVH (30). Duration of mechanical ventilation in infants with IVH was higher. In Badiei study, also, duration of mechanical ventilation time was an important IVH risk factor (26). Results of a study showed that infants who need the mechanical ventilation had 7 to 8 times more chances of bleeding than infants without this kind of treatment (27). 

In the present study, 63% of infants with IVH were boys. Mohamed co-workers showed in a study that boys with lower weight in comparison with girls are at high risk of IVH and severe IVH and association of male gender with IVH and its intensity was more powerful than birth weight (31). The prevalence of respiratory distress syndrome in infants with IVH was 68.05% and in the control group, this was 19.70%. Levene co-worker’s showed that if respiratory distress syndrome was along hypercapnia and severe acidosis, it would have a relationship with IVH (32). In Abdi and co-worker’s study, the relationship between RDS and IVH was not approved (25). In Badiei and co-worker’s, RDS was in 53 infants in which 13 infants (24.5%) hade IVH and the frequency of IVH in terms of RDS was not significant, according to the statistic (26). In infants with severe RDS who were undergoing high mechanical ventilation, increasing cerebral vein pressure or velocity fluctuation in cerebral blood flow and IVH risk will be increased (33). 

Hospital control group which was inevitable was considered as one of the limitations of the present study. The results of the present study indicated that about four-fifth of the premature infants, suffered from vitamin D deficiency and approximately one-third suffered from severe vitamin D deficiency. In infants with IVH, serum vitamin D mean was lower and infants with RDS, mechanical ventilation and vitamin D deficiency being susceptible to the high IVH risk. 

## Conclusion

Our study indicated that about 93% of infants with IVH had vitamin level below 30 ng/ml, while in infants without IVH this was 67%, therefore according to the existence of an inverse relationship between serum vitamin D level and IVH occurrence, vitamin D deficiency may have a role in IVH occurrence.
